# MindSurf: a pilot study to assess the usability and acceptability of a smartphone app designed to promote contentment, wellbeing, and goal achievement

**DOI:** 10.1186/s12888-016-1168-z

**Published:** 2016-12-12

**Authors:** Timothy A. Carey, Jennifer Haviland, Sara J. Tai, Thea Vanags, Warren Mansell

**Affiliations:** 1 Centre for Remote Health, a joint centre of Flinders University and Charles Darwin University, PO Box 4066, Alice Springs, NT 0871 Australia; 2 School of Psychological Sciences, University of Manchester, Manchester, UK; 3 Birtinya, QLD Australia

## Abstract

**Background:**

The Method of Levels (MOL) is a transdiagnostic cognitive therapy that promotes contentment, wellbeing, and goal achievement through the resolution of internal conflicts underlying psychological distress. MOL, based on Perceptual Control Theory (PCT), was developed in routine clinical practice and has been used effectively across different health services by different practitioners. Access to MOL-style questions through a smartphone app could, potentially, help both the general public maintain robust mental health, and also be a useful adjunct to therapy for clinical populations. The app is called MindSurf because of its focus on helping people explore their thinking. Prior to developing the app and using it with different populations it was necessary to determine whether such an idea would be usable for and acceptable to potential app users. Therefore, a pilot study was conducted with a non-clinical sample to assess the usability and acceptability of the app including monitoring whether the questions delivered in this way were associated with any adverse events.

**Methods:**

A pilot study using quantitative as well as qualitative methods and incorporating a repeated measures, A-B design was conducted.

**Results:**

The 23 participants were healthy adult volunteers who were all either undergraduate students, postgraduate students, or staff of the University of Manchester. They received MOL-style questions on their mobile phones over a 1-week period. Qualitative results were encouraging and indicated that the format and style of questioning were acceptable to participants and did not lead to increased worry or concern. A one-way, repeated measures ANOVA indicated that there was a nonsignificant decrease in scores on the 21-item Depression, Anxiety, and Stress Scale (DASS21) over a 2 week period.

**Discussion:**

The results of the pilot study justified development of MindSurf and further testing once it is available for use. A power analysis indicated that the pilot study was underpowered to detect significant effects but provided important information regarding the appropriate sample size for future research. The pilot study also indicated that future research should investigate the effects of receiving more than three questions per day.

**Conclusion:**

Results of the pilot study indicate that MindSurf will be a usable and acceptable app. Its benefits should be further explored through longer studies with larger sample of both the general population as well as clinical populations.

## Background

Mental health problems are a growing international concern. Approximately half of all ill-health in UK residents under the age of 65 is due to mental illness [1] accounting for 23% of the total burden of disease [2]. In order to minimise the impact of mental health problems, gaining timely access to effective psychological interventions is imperative. It could also be argued that, just as important as improving access to therapy for people experiencing mental health problems, is the development of preventative approaches that are useful for the general public in diverting some people away from developing problems that need formal therapy to ameliorate.

The rise of the smartphone has been astonishing with an estimated 1.82 billion smartphones in active use worldwide by the end of 2013 [3]. A recent survey indicated that smartphone users are with their phones for all but 2 h of every day [4]. Importantly for this research, other studies have indicated that 25% of adults use apps for health care [5] and 71% of patients in a psychiatric outpatient setting indicated a desire to use apps as an adjunct to their clinical care [6].

Smartphones have been described as revolutionizing approaches to wellbeing investment [3]. Links between smartphone technology and health outcomes have been demonstrated in many fields with preventative health and clinical outcomes being at the forefront [3]. Smartphone interventions, for example, have had both preventative and management benefits in relation to health [7] and psychological treatments via smartphones can augment the effects of face-to-face therapy sessions by providing opportunities for individuals to engage in homework tasks and practice skills in between sessions [8].

Despite the potential benefits of smartphone applications there is a concerning lack of regulation and an absence of evidence for many of these applications [3]. Although there are more than 15,000 health related apps available, very few of them have been assessed through research [9]. While apps for mental health problems are potentially effective, and can dramatically improve treatment accessibility, the majority of apps lack any scientific evidence about their efficacy [10, 11]. In one study only 2.6% of apps for depression made any attempt to substantiate claims of effectiveness [11]. Furthermore, recent research [11] has demonstrated that the mental health app marketplace is volatile in terms of how rapidly app availability can change which may erode confidence in the ability of these resources to provide reliable and effective support. Therefore, while smartphones are ideal tools in many ways for providing instantly accessible and destigmatizing health promotion and intervention resources [12], it is imperative to ensure that the resources are based on systematic research in order to demonstrate they are not simply placebos or, indeed, harmful [3]. As with other research [12], an important starting point in app development would seem to be an assessment of the usability and acceptability of the concept to potential app users.

## Applying a cognitive therapy to a mobile phone

The Method of Levels (MOL) [13–16] is a transdiagnostic cognitive therapy based on the principles of Perceptual Control Theory (PCT) [17]. MOL synthesises the three core processes of control, conflict, and reorganisation into an efficient and effective treatment modality [16, 18]. The questioning style used in MOL promotes a detailed exploration of cognitive processes and, in particular, the way in which different cognitive states relate to each other. MOL has been developed and evaluated in routine clinical practice in both Australia and the United Kingdom since 2002 with effect sizes such as 0.77, 1.36, and 1.45 reported [18, 19].

Apart from the demonstrated effectiveness of MOL [18, 19], one of the interesting clinical outcomes that has been reported anecdotally by patients is that, through repeated MOL sessions, patients begin to internalise the MOL questioning style. Since one of the priorities of MOL is to help the patient to “get along without the therapist”, it was of interest to determine if technology could be used to provide a resource for people to access MOL-style questions outside therapy sessions. Indeed, given how helpful patients report these questions to be, it was also of interest to determine if these questions would be useful for non-clinical populations.

It was planned to develop a mobile phone app that would push MOL-style questions at random times throughout the day to the user’s phone. The number of questions would be set by the user as would the time period within which the questions were delivered. The user would be prompted to either type an answer or to say an answer out loud, because in MOL it is considered that the external expression of internal cognitions is a useful way to promote greater depth of processing [16].

Given the importance of ensuring that the app is evidence-based, a systematic program of research was developed (see Table 1). Prior to developing the app it was of interest to determine how such an app would be experienced. Sometimes the questions in MOL can be initially unsettling for patients as they grapple with incongruities and dissonance in their belief systems [16]. Initially a proof-of-concept exercise was conducted with one person over the period of 1 week to assess the feasibility of the idea. The proof-of-concept exercise demonstrated that the concept was feasible and did not necessarily create irritability or stress by receiving the questions. Next, a pilot study was conducted over 2 weeks with 23 participants to assess the usability and acceptability of the concept as well as monitoring the possibility of adverse events.

**Table 1:** MindSurf research development plan

Stage	Research	Study Characteristics
I	Proof-of-concept exercise	*n* = 1; short time frame; manually send questions
II	Pilot study	small n; non-clinical sample; short time frame; assess usability and acceptability, mixed methods; manually send questions
III	Wellbeing, contentment, and goal achievement study	large n; non-clinical sample; population based study; extended timeframe; assess impact of app on wellbeing, contentment, and goal achievement in the general population; mixed methods randomised controlled trial; smartphone app developed and used
IV	Impact on psychological distress study	large n; clinical sample, extended time frame; assess impact of app on reduction of psychological distress; mixed methods randomised controlled trial comparing face-to-face individual therapy without the app and face-to-face individual therapy including the app; smartphone app used

## Aims, research questions, and hypothesis

The aim of the pilot study was to assess the usability and acceptability of receiving MOL-style questions via intermittent daily text messages. It was also of interest to assess whether sending MOL-style questions that can sometimes be confronting in therapy would be associated with adverse events when receiving them as text messages on a mobile phone. Specifically, two research questions guided this pilot study:

- RQ1 How do participants describe their experience of receiving and responding to MOL-style questions received throughout the day via text messaging?
- RQ2 Do people who receive MOL-style questions via text messaging throughout the day report changes in psychological distress?

The first research question required a qualitative methodology to answer whereas the second question used quantitative methods. For RQ2 an hypothesis was generated:

- H1 Participants will show improvements in psychological distress as evidenced by statistically significant decreases in scores on the 21-item version of the Depression Anxiety Stress Scale (DASS-21) [20].

For RQ1, semi-structured interviews were used. The usability and acceptability of the app was determined by participants’ responses in the interviews. Also, completing the week of responding to three text messages a day was used as a behavioral indicator of the usability of the app.

## Methods

With regard to the methods of the study, this paper conforms to COREQ guidelines ((http://intqhc.oxfordjournals.org/content/19/6/349.long).

### Participants

Participants were a self-selected, convenience sample recruited through an online volunteering database at the University of Manchester and in person by the second author (JH). The volunteering database is a website where all research projects for the University of Manchester that require research participants are listed. Volunteers are then able to select studies in which they are interested in participating and establish contact with the researcher for more information. Of the 23 people who volunteered, three were male and all were over the age of 18. A large proportion of female participants is not unusual in these types of studies [21] and is likely to be because the majority of participants were psychology students who are predominantly female.

Information about the rationale for the study and what would be required of participants was displayed on posters around the University of Manchester. This information was also available at the online database where people could indicate their willingness to participate in the research. Furthermore, all participants were provided with a standardised Participant Information Sheet prior to consenting to participate in the research. Given that the focus of the study was usability and acceptability, other potentially useful measures such as assessing the roles of expectancy or credibility were not used but may be included in future research. Participants were aware, however, that they would be asked to participate in an interview at the end of the study which would explore their experience of receiving the questions. A small monetary incentive of £5 was offered to participants upon completion of the 2-week data collection period.

As with other studies assessing the usability and acceptability of smartphone apps, inclusion and exclusion criteria were minimal [12]. Individuals who were not fluent in English were exempt from participation due to the study being dependent upon written and verbal comprehension. Participants were also required to be in possession of a mobile phone in order to receive text messages throughout the duration of the study. None of the people who indicated an interest in participating in the research were excluded and no participants withdrew from the study.

### Materials

#### Depression Anxiety and Stress Scale 21 (DASS-21)

The DASS-21 [20], a 21-item measure derived from the original 42-item self-report measure, was used to assess participants’ level of psychological distress. Samples completing the DASS-21 demonstrate adequate psychometric properties for the measure [22] and some research indicates that the 21-item DASS is superior to the 42-item version in terms of its factor structure [23].

The DASS-21 [20] requires responses to 21 statements on a 0 (“Did not apply to me at all”) to 3 (“Applied to me very much, or most of the time”) scale. Scores can range, therefore, from 0 to 63 with higher scores indicating greater levels of depression, anxiety, and stress.

#### Diary booklets

Participants were provided with two paper-copy diary booklets for completion, one for each week of data collection.

##### *Pre app* booklet

The diary booklet for week one consisted of two 10-point Likert scales to be completed once daily; one indicating the perceived levels of wellbeing and the other indicating perceived levels of distress. For the perceived wellbeing scale, a score of zero indicated an extremely low level of wellbeing and a score of ten indicated an extremely high level of wellbeing. For the perceived distress scale, a score of zero indicated “not distressed at all” and a score of ten indicated “extremely distressed”.

##### App booklet

The diary booklet for week two prompted participants to write the responses to the questions they were sent via text message in the text boxes provided (three per day). As in week one, participants were also requested to record once daily their perceived levels of wellbeing and distress (using the same Likert scales used previously). Participants were asked to record these ratings after having received all the questions for the day.

#### Recording devices and software

Interviews were recorded using a digital dictaphone device. Both SPSS (Version 20) and NVivo (Version 10) were used in the analysis of the data.

#### Question bank

A bank of 52 questions was developed by the first author (TAC) based on the types of questions asked in MOL. The questions are designed to help people increase their awareness of their current cognitive activity. Some of the questions include a pair of questions to draw people’s attention to various cognitions and then have them reflect on that. The question bank includes questions such as: *“What’s going through your mind just at the minute?”; “What’s your perspective on things right now? What’s the thought that stands out the most?”; “What goal are you heading for right now?”; “What is going through your mind at this very minute? What do you make of that?”;* and *“Where’s your attention just now?”*

### Intervention

The intervention for this study was the app described in the introduction. The essential feature of this app is that it pushes to a user’s phone questions, in the form of text messages, which encourage metacognitive thinking. These questions have been found to be therapeutically useful in clinical situations. The concept of the app is innovative in that it pushes questions at random times throughout the day. Without any evidence of usability or acceptability it was considered premature to develop an actual app so the researcher functioned as the app and sent three text messages to the participants’ phones at random times throughout the day over a 1-week period. If the results of this study indicate that the development of an app should proceed, the number of questions (between 0 and 10) will be set by the user as will the time period each day within which the questions will be sent (e.g., start at 7.00 am and finish at 6.00 pm).

### Procedure

Ethics approval was obtained from the University of Manchester Research Ethics Committee prior to starting the study. Participants were informed of the nature of the study and the content of the questions they would receive (i.e., reflecting upon thoughts and feelings) before consent was obtained. Participants were advised to only provide that amount of information in response to the questions which they felt comfortable sharing.

#### Phases of the study

Table 2 provides a summary of the four phases of the A-B design of the study. In the baseline phase participants completed the DASS-21. The pre app phase lasted 7 days. During this time participants rated both their subjective wellbeing and psychological distress on a scale ranging from 0 (indicating the absence of wellbeing or distress) to 10 (indicating extremely high levels of wellbeing or distress). At the end of the 7 day pre app period, participants once again completed the DASS-21. In the 7 day app period, participants received three MOL-style questions at random times each day via text messages. Upon receipt of the question, participants were invited to record an answer to the question. During these 7 days participants continued to rate their levels of wellbeing and distress. Following the app phase of the study, participants once again completed the DASS-21. Additionally, participants were invited to attend a short, semi-structured interview in which questions were asked about their experience of receiving and answering the text messages throughout the app phase.

**Table 2:** The four phases of the pilot study describing the tasks involved at each phase

Baseline	Pre App	App	Post App
Complete DASS-21	Rate subjective wellbeing and distress daily for 7 days	Rate subjective wellbeing and distress daily for 7 days	Complete DASS-21
	Complete DASS-21 after 7 days	Record answers to MOL-style questions received as text messages	Attend short, semi-structured interview to describe the experience of receiving and answering questions

#### Procedure for analysis of interview data

The semi-structured interviews were recorded and then transcribed. A topic guide consisting of five core questions to explore usability and acceptability was used to structure the interviews. The questions were: What was your experience of receiving the questions daily via text message?; What did you think of the questions themselves?; Did you find the questions helpful at all? Why?; Did the questions help you realise anything? What was this?; Would you have preferred more or fewer questions each day? Why is this? From these questions, additional probes were used to examine responses in greater detail.

Thematic Analysis (TA) was used to identify recurring themes in the data obtained from the transcripts. The initial stage of TA was familiarisation with the data including reading the interview transcripts several times in order to increase sensitivity to emerging themes. The data were systematically coded with attention focused on recurrent content and content relevant to MOL and PCT. These codes were then grouped and organised into subthemes which, when reviewed and analysed, were regrouped under broader, conceptual themes. All subthemes and themes were reviewed in an iterative process and subthemes that did not have sufficient recurrent, representative data were discarded.

### Design

A repeated measures, A-B design was used for this study. Data were collected over a 2 month period because participants completed their 2 week engagement in the study at different times depending on their availability and time of recruitment.

## Results

### Quantitative analysis

The quantitative data and analysis, for the purposes of this study, indicated potentially adverse effects and should be regarded as an addition to the qualitative data which explores the usability and acceptability of the app. Scores from the DASS-21 at the three time-points of baseline, pre app, and post app were analysed using a one-way, repeated measures analysis of variance (ANOVA). Repeated-measures ANOVAs were also conducted on daily ratings of perceived wellbeing and distress which were obtained over a 14 day period.

#### Hypothesis one

It was hypothesised that the MOL-style questions would result in significant decreases in scores on the DASS-21. The means and standard deviations for the DASS-21 at three different time points (baseline, pre app, and post app) are presented in Table 3. There was a decrease between the first and last mean DASS-21 scores and the difference between the mean scores at the three time points approached significance *F*(2, 25) = 3.10, *p* = 0.06. This trend was encouraging and suggests that adverse events do not seem to be indicated.

**Table 3:** Means and standard deviations for the DASS-21 as well as the subjective ratings of wellbeing and distress at three time points

Measure	Time 1		Time 2		Time 3	
	Mean	*SD*	Mean	*SD*	Mean	*SD*
DASS-21	12.18	6.78	9.23	4.22	9.45	5.33
^a^Distress	2.90	1.80	2.80	1.60	2.60	2.30
^a^Wellbeing	6.80	1.50	7.10	1.30	7.30	1.20

^a^Wellbeing and distress ratings were collected over 14 days. In this table the ratings at Day 1, Day 8, and Day 14 are provided

#### Daily ratings

Daily ratings of wellbeing and distress were obtained from participants over 14 days (7 days prior to receiving the questions and 7 days while receiving the questions). The mean ratings for Day 1, Day 8, and Day 14 are provided in Table 3. The ratings remained relatively constant across the 14 day period indicating that the introduction of the questions did not overly perturb or bother the participants. The pattern of these mean ratings provides further evidence that converges with the DASS-21 scores to suggest that adverse events do not seem to be indicated with receipt of the MOL-style questions.

### Power analysis

Given that the direction of change for the DASS-21 was in the desired direction, a power analysis was conducted to determine if the failure to achieve statistical significance was due to the study being underpowered. With a sample size of 23, the alpha level set at 0.05, and an effect size for the DASS-21 of *η* ^2^ = 0.20 respectively, the power for this study was 0.52. To increase power to 0.95 a sample size of 66 is required.

### Qualitative analysis

Of the 23 participants in the study, 11 consented to participate in semi-structured interviews. The most common reason participants gave for not participating in the interview was a lack of time available in their busy schedules. Table 4 lists the themes and subthemes derived from the interviews.

**Table 4:** Themes and subthemes of participants’ reported experiences of receiving the MOL-style questions

Themes	Subthemes
Perceptions of the app	Modality Helpfulness
Convenience	
Comprehension	
Enhanced awareness	Present-moment thinking Self-reflection Barriers to self-reflection

#### Perceptions of the app

Analysis of the transcripts revealed that all participants had varying opinions regarding the app and differential experiences of receiving the questions. All interviewed participants recognised the potential benefits of receiving the questions and engaging in a psychotherapeutic intervention via technological modalities, although there were differing views of how relevant such interventions were to their everyday experiences. Interviewees clearly expressed their perceptions of engaging in interventions via mobile phone technology and their degree of willingness to engage in such interventions via technological modalities.

#### Modality

When questioned regarding the acceptability of using mobile phones for such interventions, the majority of participants thought that mobile phones were acceptable and convenient. Participants reported that they were able to integrate a mobile phone-based intervention into their everyday lives relatively easily due to mobile phones being integral to daily routines:

*‘Yeah, I thought it was good … text messages suited me fine … it’s good to text you, like, at random times to catch you in the moment.’ [103]*

*‘… it was quite good to be honest because most people, y’know, keep their phone with them at all times so, like it was something I’d do right there and then.’ [109]*

#### Helpfulness

Participants recognised the potential benefits of the app and were able to identify ways in which it could be personally helpful:

*‘… some of them, like, actually made me just think more about the bigger picture so that was quite positive.’ [113]*

Also, participants identified particular circumstances in which the questions would be helpful such as when they feel their wellbeing is compromised or their distress levels are increasing.

*‘I think it depends on the situation you're in at the time as well - because if you're in a situation that's either stressful or quite demanding - the way you're thinking at that time and the question you receive whether it's helpful or not I think can have a big difference on…how it would make you cope later on … the questions I think mostly would all be really helpful just depending on what kind of circumstances you’re under at that time.’ [104]*

#### Convenience

Participants emphasised how important it was to be able to incorporate the app into their everyday lives in terms of the usability and acceptability of the approach.

*‘… it took up quite a bit of time … well, it didn’t take up time it was just, erm, hard to answer them when I got them…’ [102]*

*‘… at times they were a bit inconvenient I think just because of obviously what I was doing at the time …’ [104]*

#### Comprehension

Some participants expressed difficulty at understanding what particular questions were asking about or at generating answers to questions. Three participants felt that this was due to the vague nature of the questions, but two participants expressed difficulty in generating an answer to the questions which they deemed sufficient.

*‘… some of them, most of them, were quite straightforward and quite easy to answer but there were some of them that were quite hard to answer…because you weren’t quite sure what it was actually asking you about.’ [102]*

*‘I just thought it was a bit, it was a bit too vague … so I didn’t really know … what the questions were asking.’ [105]*

#### Enhanced awareness

All participants identified and acknowledged that the questions received via text messages enhanced their self-awareness to varying degrees. Participants reported individually that the MOL-style questions prompted them to enhance their awareness of issues such as their thought patterns, their personal goals, and their behavioural responses. The overarching theme of enhanced awareness could be clearly separated into the subthemes of: present-moment thinking, self-reflection, and barriers to self-reflection.

#### Present-moment thinking

Participants made reference to the fact that the questions prompted them to consider their present-moment thoughts and thought patterns, potentially allowing them to adopt a more mindful approach to their emotions and cognitions. For example:

*‘[The questions] made me more aware in that moment of the way I think …’ [101]*

*‘I think doing the questions … made me think about – how to be more aware of my thoughts … it sort of … made me think about thinking about my thinking if that makes sense?’ [103]*

#### Self-reflection

Participants reported that receiving the questions prompted reflection. This reflection appears to have often been inwardly directed, thus resulting in participants becoming increasingly self-aware and reflecting on patterns of thought and behaviour which may play a general role in their wellbeing.

*‘… it enables you to reflect on what you’re thinking and then … kind of check yourself and be like ‘I shouldn’t be really thinking that’ so that was quite good I think …’[101]*

*‘… it was nice to sort of think about what I’m doing and whether it makes sense or not.’ [105]*

#### Barriers to self-reflection

Despite participants reporting that the questions and the app as a whole enabled them to be more self-reflective and more aware of patterns of thoughts, behaviours, and emotions, participants also reported sometimes experiencing difficulty engaging in the activity of self-reflection. This emerged as a separate issue to the comprehension of the questions, but instead appeared to revolve around having difficulty capturing and analysing current thoughts.

*‘… if you’re busy on a particular, sort of, job or task or whatever where you’re preoccupied by things where you’re … preoccupied by the task and not preoccupied by other thoughts.’ [103]*

*‘… a lot of the time it would just be like, well I’m not really thinking anything right now. I was just like not really doing anything and my mind was pretty blank.’ [108]*

## Discussion

Given the increasing burden of mental health problems and the difficulty in accessing effective services in a timely manner, a pilot study, preceded by a proof-of-concept exercise, was conducted to investigate the usability and acceptability of a smartphone app. The app is based on MOL, a transdiagnostic cognitive therapy. The proof-of-concept exercise indicated that the questions were received favourably across a 7 day period and that some useful realisations were achieved. The focus of this paper, the pilot study, was similar to other projects investigating the use of apps in that it had a small, self-selected convenience sample, was conducted over a relatively short time-frame, and focussed on usability and acceptability [3, 12]. The pilot study demonstrated that, across the data collection period, receiving the questions was not associated with adverse events. Rather, participants generally engaged well with the questions.

From the qualitative data in the pilot study the themes of: “perceptions of the app” (with subthemes of “modality” and “helpfulness”); “convenience”; “comprehension”; and “enhanced awareness” (with the subthemes of “present-moment thinking”, “self-reflection”, and “barriers to self-reflection”) were generated. Participants indicated that, generally, they found the questions helpful in the way that was intended. That is, they became more aware of their thought processes and more reflective. Participants also reported finding some of the questions difficult to answer, but found the modality of a mobile phone convenient. It could be that, when the app is used an adjunct to MOL therapy, or even over a longer time frame, the questions would not be vague because people would be familiar with them. The qualitative data, therefore, indicate that the app is both usable and acceptable.

Although there is a paucity of research about health related apps [9], the results of this pilot study compare favourably with other studies that have been conducted. A study of a mindfulness app using a short time-frame (10 days), a self-selected sample, standardized questionnaires, and a randomized controlled trial design reported significant effect sizes of 0.071 for positive affect and 0.03 for depression [3]. The effect sizes for flourishing, satisfaction with life, and negative affect were non-significant [3]. Also, a study over a 2-week time frame investigating the use of a music app for emotion regulation used the 42-item version of the DASS to assess emotional health [24]. Subscales of the DASS were correlated with various antecedent-focused and response-focused strategies with mixed results. Of the 39 correlations, 15 were significant [24]. Another pilot study used a small (*n* = 10), convenience sample with no inclusion criteria to investigate the usability and acceptability of an app to promote wellbeing in a clinical population [12]. As with our pilot study, this study report a high rate of engagement with the app.

The quantitative data did not demonstrate a significant reduction in psychological distress although the focus of this pilot study was on assessing the usability and acceptability of the app and the function of the quantitative data was to indicate the possibility of adverse events. These pilot study data indicate that adverse events seem unlikely. In fact, given the way in which receiving the questions was experienced by the participants, we can have some confidence when developing the app that the questions are unlikely to excessively irritate or distress the app users. To investigate reductions in psychological distress it would seem that the lessons from this pilot study are that a clinical sample would need to be used in a research program over a longer period of time and perhaps receiving more than three questions per day.

It was encouraging that even over the period of 7 days, receiving just three questions per day, the participants reported increases in awareness and self-reflection. If the app was developed to allow users to schedule more questions per day and to receive the questions over longer periods of time, then greater benefits may be observed. Using larger samples with varying levels of wellbeing and distress will provide more information on the benefits of the app for different populations.

### Limitations

Even though small sample sizes are appropriate for pilot studies, the sample size does limit the conclusions that can be drawn. Moreover, the power analysis indicated that the pilot study was underpowered to detect the effect of the app. Also, the impact of the questioning may have been restricted given that only three questions per day were sent to participants’ phones, and this occurred only over a 7-day period. Sending three text messages per day to 23 people can be an onerous task when done manually, however, when the app is developed, the app users will be able to specify the number of questions to receive each day providing the flexibility that allows different users to suit their individual needs.

Another limitation may be that only approximately half of the sample participated agreed to be interviewed. This may have introduced a selection bias whereby it was those participants who experienced the questions positively who agreed to be interviewed. People who did not participate in the interviews most commonly gave the explanation of being too busy and did not indicate that they had experienced the questions negatively, however, this remains an empirical question that could be clarified in future research.

### Future research

The results of the pilot study have justified development of the app. Because of the focus on exploring the contents of one’s mind, the app will be called MindSurf. Once MindSurf has been developed it will be possible to conduct further studies with large sample sizes over longer periods of time and with both non-clinical and clinical populations. As indicated in Table 1, a large scale study to investigate the ability of MindSurf to promote contentment, wellbeing, and goal achievement in the general population will be important as will an examination of the ability of MindSurf to assist in reducing psychological distress in clinical populations. Larger scale studies will also make it possible to measure other variables such as the roles of expectancy and credibility.

From the qualitative analysis in this study it was evident that some participants found one or more of the questions difficult to understand. In future research it will be possible to explore this lack of understanding in greater detail and to revise and improve the questions where necessary. It could be that if people use the app over a longer period of time with a greater range of questions that the questions in general will become more comprehensible. Participants themselves may also be able to offer questions that could be included in the question bank.

The post hoc power analysis that was conducted has provided useful information regarding the sample size required in future research (n = 66). It will also be possible in future research to investigate the impact of question frequency on effect size. It may be that, with an increase in questions per day from 3 to, perhaps 10, that a larger effect size would be obtained.

Given the number of mobile phone apps available now that address different aspects of mental health, it would also be possible to use research designs that incorporated comparison groups. As well as comparing MindSurf to other apps, it will also be of interest to ascertain whether MindSurf enhances the effects of therapy by comparing a therapy as usual group with a therapy plus MindSurf group. Despite the availability of wellbeing apps, there is a startling lack of evidence for most of them [9] and the feature of sending MOL-style questions at random times throughout the day appears to be unique and provides further avenues for research such as investigating whether varying the number of questions received has an impact on contentment, wellbeing, and goal achievement. With the MindSurf app it will also be possible to examine more closely the way in which the MOL questions exert their therapeutic benefit. Possible questions for future research could be: Do people who internalize the MOL questions experience greater benefits than people who don’t?; and Are some questions more helpful in certain circumstances than other questions?

## Conclusions

The results of this pilot study indicate that MindSurf will be an acceptable and usable app that does not appear to generate adverse events. Further research is warranted to investigate the use of the app as a way of promoting wellbeing in the general public and also helping to reduce psychological distress in clinical populations. The unique feature of generating questions randomly throughout the day to enable the examination of a person’s cognitive processes “in the moment” may prove to be an important factor in experiencing sustained and robust contentment and satisfaction and lasting reductions in psychological distress. The systematic development and evaluation of MindSurf is providing an evidence-based addition to the large number of health and wellbeing apps already available and helping to address the urgent priority of providing timely access to effective mental health support.
